# Pictures over words: a cross-sectional study reporting short term memory abilities in children

**DOI:** 10.12688/f1000research.23378.3

**Published:** 2021-01-08

**Authors:** Aysha Rooha, Malavika Anakkathil Anil, Jayashree S. Bhat

**Affiliations:** 1 Department of Audiology and Speech-Language Pathology, Kasturba Medical College, Mangalore, Manipal Academy of Higher Education, Manipal, Karnataka, 575001, India

**Keywords:** Cognitive communication, Recall, Short Term Memory, Stories

## Abstract

**Background:**  An impressive amount of research has been conducted studying the modality effect on multimedia information in children from higher elementary school to college. In the present study, we aimed to examine the modality effect in the recall of multimedia information among children between the age range of 6 years to 9 years 11 months.

**Methods:** The study followed a cross-sectional design and comprised of 80 participants between the ages of 6 years to 9 years 11 months. An animated story was shown to the children, following which a word recall task was performed. In this task, children were asked to recall the words mentioned in the story from a pictorial array.

**Results:** One-way analysis of variance revealed a significant difference in the overall recall abilities of children. The recall performance was strongly related to the modality of the presentation of words. A marginal difference was observed for the recall of auditory-visual words in comparison to recall of words in the auditory modality; wherein older children recalled better in comparison to younger children. The findings of the study could be attributed to the "visual superiority effect", "encoding specificity principle of memory" and "multimedia effect."

**Conclusion**: Recall abilities were observed to increase with age, with the existence of asynchrony in the auditory-visual and auditory recall scores indicating the firm reliance on the modality of presentation of word. The study implications emphasize on the use of visual stimuli for teaching new vocabularies, skills, and concepts in younger children. These findings also highlight the use of visual stimuli while assessing speech, language, and cognitive skills in younger children.

## Introduction

Short term memory (STM) skills are dependent on the modality of presentation of stimuli ( Goolkasian & Foos, 2002; Paivio *et al.*, 1975). Research on short term memory skills has been examining the effect of bimodal and unimodal presentation of information on the recall performance. Frick (1984) studied the effect of modalities on digit span duration task and found superior span duration for bimodal presentation when compared to unimodal. Pillai & Yathiraj (2017) studied the effects of modality on four different memory skills (memory score, sequence score, memory span and sequence span). The study revealed the effect of modality only for the memory score. It was observed that memory scores were higher for auditory modality and combined (auditory-visual) modality when compared to visual modality, with no significant difference between the auditory and combined modality.

Similarly, research exists on the modality effect in multimedia learning. Moreno & Mayer (1999) found that students performed better on retention, transfer and matching tests when the auditory stimuli (narration) were combined with the visual stimuli (texts or animation) rather than presenting the visual stimuli alone. Various other studies ( Brünken *et al.*, 2004; Mayer & Moreno, 1998; Mousavi *et al.*, 1995) have also supported the notion of modality effect in multimedia learning. The modality effect observed is supported by the cognitive theory of multimedia learning ( Mayer, 2001), which states that presenting information through two channels reduces cognitive overload as the working memory capacity is limited. However, most of these studies focus on college students and little is known about the modality effect on multimedia learning in school going children.

Mann *et al.* (2002) using an experimental design studied the modality effect on 12-year-old children, where participants were assigned into one of the two conditions (animations with visual information or animation with auditory information). The study did not find any difference between the two experimental conditions revealing absence of modality effect in children. Witteman & Segers (2010) assessed the modality effect in sixth-grade children (mean age 11.8 years). The participants were assigned to either of the two conditions (pictures with auditory condition or pictures with text condition) and were assessed for retention and knowledge transfer. The evaluation was done immediately after intervention, the following day and a week later. The study did not find any modality effect in children, rather found a reverse modality effect for retention directly after the intervention, and for transfer questions one day later. Research on modality effect in multimedia information in children has focused mostly on children from higher elementary years.

In the present study, the authors explored the modality effect in multimedia information among primary school children. With computers and digital media starting to gain momentum in the educational system, the finding of the present study may help identify whether using multimedia information is advantageous or not for young children. The present study aimed to examine the modality effect in recall of multimedia information among children between the age range of 6 years to 9 years 11 months.

## Methods

### Study design

The study followed a cross-sectional design that was approved by the Institutions Ethics Committee (Ethical Reference Number - IEC KMC MLR 11-18/463). The study was conducted between December 2018 and January 2020. The study was conducted within the classroom setting of the approached school. Written informed consent was procured from parents of children who agreed to take part in the study.

### Participants

English medium schools affiliated to the Central Board of Secondary Education (CBSE), Mangalore city, were approached, after obtaining authorization from the Block Education officer, to recruit participants for the study. Those schools that provided permission to conduct the study were considered for data collection. Typically developing children who passed the WHO Ten-Question Disability Screening Checklist ( Singhi *et al.*, 2007) were recruited for the study. Children with a history of any transfer from more than one school; a history of any shift in the medium of instruction; or a history of academic failures were excluded from the study.

A sample size of 80 was determined with respect to the study done by Appose & Karuppali (2018) using the formula: n = Zα ^2^σ ^2^/d ^2^ where, Zα = 1.96 at 95% confidence level, d = 20% of the mean and, σ = standard deviation. The 80 participants were assigned equally into four groups (Group I: 6 years – 6 years 11 months; Group II: 7 years – 7 years 11 months; Group III: 8 years – 8 years 11 months; and Group IV: 9 years – 9 years 11 months ).

### Data collection

A story, “The Wooden Box” (copyright © 2019, Anil and Bhat), was constructed as animated stimuli and a “Word Recall” task ( *Extended data* ( Rooha *et al.*, 2020)) was formulated based on the story to assess the recall ability. The final modified task included 12 pictorially represented words that had an equal number of *words from the story* (Gold coins, Cupboard, Keys, Traffic), *words thematically related* (Hut, Chair, Diamond, Bag), and *words unrelated to the story* (Apple, Chair, Frock, Flower). Among the four *words from the story,* two of the words were presented in the auditory-visual modality (Gold coins, Cupboard), while the other two words were presented in auditory modality alone (Traffic, Keys). Each of these words appeared only once in the story. The task was to identify the pictures representing the words from the story. The recall accuracy was calculated by giving a score of 1 for each word correctly recalled. 

The animated story as well as the formulated task was content validated by three speech-language pathologists and three primary school teachers of CBSE. The suggestions provided included using Indian names for the characters of the story, to modify the instructions and to modify certain words in the formulated task. These suggestions were incorporated in the preparation of the final stimuli.

Each child was evaluated individually in a classroom. The animated story was presented on a laptop screen, immediately followed by the administration of the word recall task. The examiner presented the task verbally, and the child’s responses were scored simultaneously.

### Data analysis

The responses were subjected to statistical analysis using SPSS software version 16.0 and significance was set at the 0.05 level (p<0.05). Descriptive statistics were used to obtain the mean and standard deviation of the data. A frequency measure was done to analyse the percentage of children recalling each of the words from each of the groups. One-way analysis of variance (ANOVA) test was used to test the difference in recall performance across the age groups. The Bonferroni post-hoc test was done to assess the pair-wise differences in performance between the groups. Further, Pearson correlation test was done to assess the relationship between the recall performance and age.

## Results

**Figure 1.**
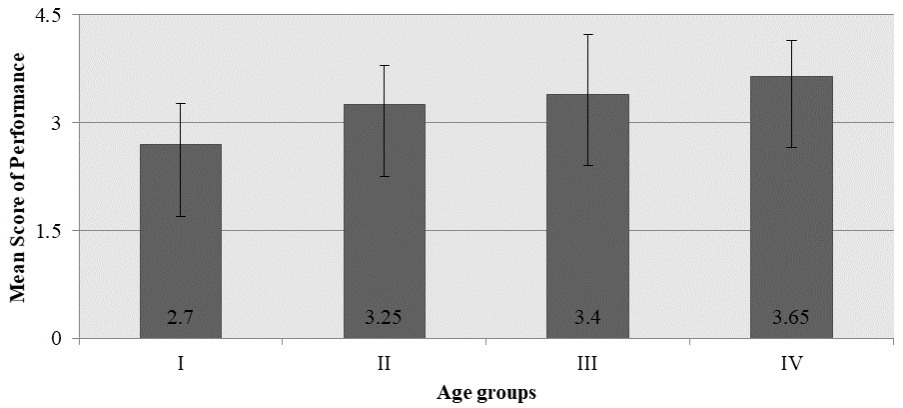
Mean scores of performance in word recall task across primary school-aged children. Group I – 6-6.11 years, Group II – 7-7.11 years, Group III – 8-8.11 years, and Group IV – 9-9.11 years.

The results of the descriptive statistics revealed a steady increase in performance across the age groups ( Figure 1). Group IV obtained the highest scores, followed by Group III, Group II, and Group I.

**Figure 2.**
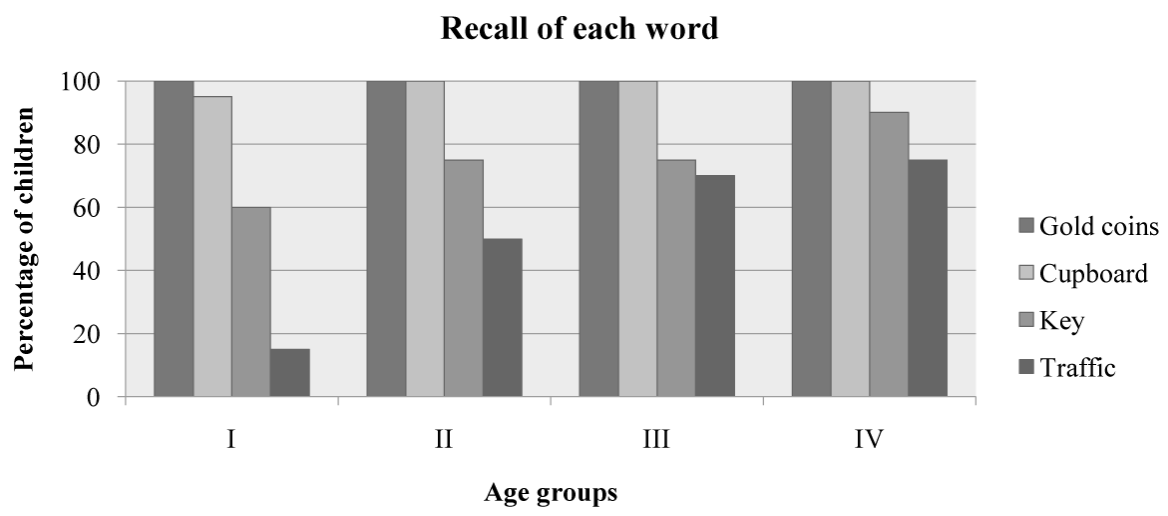
Percentage of primary school-aged children who recalled each of the words correctly in a word recall task. Group I – 6-6.11 years, Group II – 7-7.11 years, Group III – 8-8.11 years, and Group IV – 9-9.11 years.

The results of one-way ANOVA revealed a statistically significant difference in the Word Recall task [F(3,76) = 8.387, P=0.000] across the groups. Bonferroni Post-hoc results revealed that only Group I differed significantly from other groups, as depicted in Table 1. Frequency measure of children who recalled each of the words across the four age groups is depicted in Figure 2, which reveals that words ‘Gold coins’ and ‘Cupboard’ were recalled by almost all children. However, recall of words ‘Key’ and ‘Traffic’ increased with age, with drastic changes in the recall of word ‘Traffic’. Lastly, the Pearson correlation test revealed a moderate positive correlation between the recall performance and age, r (78) = 0.48, p<0.01.

**Table 1.** Post hoc results of word recall task in primary school-aged children. Group I – 6-6.11 years, Group II – 7-7.11 years, Group III – 8-8.11 years, and Group IV – 9-9.11 years.

Groups	I – II	I – III	I – IV	II- III	II – IV	III – IV
p - value	0.039 *	0.004 *	0.000 *	1.000	0.271	1.000

* Significance at the 0.05 level

## Discussion

The results revealed that the recall ability increases significantly with age. The findings agree with the study done by Belacchi *et al.* (2017), where they concluded that the word recall scores increased significantly from 6 to 12 years. While there exists an effect of age on the recall performance, the results of the Pearson’s correlation test revealed that only 40% of the time age is the contributing factor. The findings of the study can be associated to other factors. The study observed a difference in recall of the words ‘Gold coins’ and ‘Cupboard’ in comparison to the words ‘Keys’ and ‘Traffic’. The differences in the recall can be attributed to the inherent characteristic of the words, as the words ‘Gold coins’ and ‘Cupboard’ were presented in auditory-visual modalities in the story. In contrast, the words ‘Key’ and ‘Traffic’ were presented in auditory modality alone. Thus, it can be observed that auditory-visual recall was superior to auditory recall in these children. From these results, it can be considered that there exists a modality effect in recall of multimedia information in children. The findings of the present study can be attributed to various reasons which are discussed ahead.

### Visual superiority effect

Younger children are fascinated by the illustrations of the story, and focus more on visual animations in comparison to the auditory narration of the story. Attention thereby forms a critical prerequisite to encode, store, and subsequently recall information. Reduced attention to auditory information may have contributed to poorer recall of words presented in the auditory modality. Hayes & Birnbaum (1980) observed similar behaviour and termed it as the “visual superiority effect,” i.e., younger children are more inclined to “look and not listen.”

Further, there exists a difference in processing the two types of sensory information. For the items to be stored in the STM, the brain has to cognitively create ‘mental images’ of these items, which are pictorial representations of words inside one’s mind. When processing visual stimuli, the brain functions to discover a ‘mental image,’ but when processing auditory stimuli, the brain has to create a mental image of the heard word for correct recall ( Hilton, 2001). These brain functions are mediated by higher cognitive skills, which develop only with age. This could have contributed to better recall of auditory-visual words when compared to auditory words.

### Encoding specificity principle of memory

This principle states that recall of memory is optimal when the retrieval conditions replicate the conditions present when memory was created ( Tulving & Thomson, 1973). In the current study, recall of auditory-visual memory was superior because the retrieval condition duplicated the conditions when the memory was formed, i.e. children had to identify the same pictures as seen in the story. However, the retrieval of auditory stimuli did not duplicate the conditions when the memory was formed, i.e. children had to identify pictures of words heard in the story, which could have contributed to poorer performance.

### Multimedia effect

It is claimed that presenting multimedia information, i.e. presentations of material using words and pictures ( Mayer, 2002) results in deeper comprehension ( Boerma *et al.*, 2016), subsequently improving recall. This can be considered as a contributing factor for better recall of auditory-visual words.

Lastly, Ferrara *et al.* (2017) reported that detailed visual memory capacity is present in children as young as six years of age, as a result of faster maturation of visual memory than the auditory memory. This can be considered as a contributing factor for observing no differences in auditory-visual recall performance across the age group.

These evidences supports the findings of the present study; an increase in recall ability with age, the existence of asynchrony in the auditory-visual and auditory recall scores, and recall performance strongly relating to the modality of presentation of information. These findings provide implications for the use of visual stimuli while teaching new vocabularies, skills, and concepts in younger children. These findings also highlight the use of visual stimuli while assessing speech, language, and cognitive skills in younger children as it will serve as a framework for maintaining their attention while evaluating various communicative skills. The present study has limitations of including fewer words to assess the recall performance and small sample size, which could be addressed in future research.

## Data availability

### Underlying data

Harvard Dataverse: Replication Data for: Short Term Memory abilities in Children, https://doi.org/10.7910/DVN/LLGPOX ( Rooha *et al.*, 2020).

This project contains the following underlying data:

- DATA 1.tab (Analysis data with raw scores for each participant)

### Extended data

Harvard Dataverse: Replication Data for: Short Term Memory abilities in Children, https://doi.org/10.7910/DVN/LLGPOX ( Rooha *et al.*, 2020).

This project contains the following extended data:

- Word Recall task.docx (Stimulus – Formulated task)

Data are available under the terms of the Creative Commons Zero "No rights reserved" data waiver (CC0 1.0 Public domain dedication).
