# Supplementary material for: Pictures over words: a cross-sectional study reporting short term memory abilities in children
Source: F1000Res. 2021 Jan 8;9:380. Originally published 2020 May 19. [Version 3] doi: 10.12688/f1000research.23378.3 (PMC7818238; doi:10.12688/f1000research.23378.3)
Supplement: Peer review history

# Peer review: Reviewer response for version 3

**DOI:** 10.5256/f1000research.38089.r76915

**Published:** 2021-01-20

**Authors:** Raju Sapkota

**Affiliations:** 1 Vision and Eye Research Institute, School of Medicine, Anglia Ruskin University, Cambridge, UK

Is the work clearly and accurately presented and does it cite the current literature?

No

If applicable, is the statistical analysis and its interpretation appropriate?

Partly

Are all the source data underlying the results available to ensure full reproducibility?

Partly

Is the study design appropriate and is the work technically sound?

Partly

Are the conclusions drawn adequately supported by the results?

Partly

Are sufficient details of methods and analysis provided to allow replication by others?

Partly

Reviewer Expertise:

NA

I confirm that I have read this submission and believe that I have an appropriate level of expertise to confirm that it is of an acceptable scientific standard.

# Peer review: Reviewer response for version 3

**DOI:** 10.5256/f1000research.38089.r76914

**Published:** 2021-01-11

**Authors:** Ramesh Kaipa

**Affiliations:** 1 Department of Communication Sciences and Disorders, Oklahoma State University, Stillwater, OK, USA

I recommend this article for indexing.

Is the work clearly and accurately presented and does it cite the current literature?

No

If applicable, is the statistical analysis and its interpretation appropriate?

Yes

Are all the source data underlying the results available to ensure full reproducibility?

No

Is the study design appropriate and is the work technically sound?

Partly

Are the conclusions drawn adequately supported by the results?

Partly

Are sufficient details of methods and analysis provided to allow replication by others?

No

Reviewer Expertise:

Speech and Language Learning

I confirm that I have read this submission and believe that I have an appropriate level of expertise to confirm that it is of an acceptable scientific standard.

# Peer review: Reviewer response for version 2

**DOI:** 10.5256/f1000research.29224.r71334

**Published:** 2020-10-28

**Authors:** Raju Sapkota

**Affiliations:** 1 Vision and Eye Research Institute, School of Medicine, Anglia Ruskin University, Cambridge, UK

1. While the discussion is mainly around specific concepts of modality difference, nonetheless, it will be useful to understand why a larger discrepancy was noted for (gold coin, cupboard vs. traffic, key) for group I compared to group IV (Figure 2).
2. I note the following in the last para - 'These evidences supports the findings of the present study; an increase in STM skills with age,' how? The description provided in the method section does not detail about how STM was tested or tapped on. 

Is the work clearly and accurately presented and does it cite the current literature?

No

If applicable, is the statistical analysis and its interpretation appropriate?

Partly

Are all the source data underlying the results available to ensure full reproducibility?

Partly

Is the study design appropriate and is the work technically sound?

Partly

Are the conclusions drawn adequately supported by the results?

Partly

Are sufficient details of methods and analysis provided to allow replication by others?

Partly

Reviewer Expertise:

NA

I confirm that I have read this submission and believe that I have an appropriate level of expertise to confirm that it is of an acceptable scientific standard, however I have significant reservations, as outlined above.

# Response

**Published:** 2020-12-25

**Authors:** Malavika Anil

**Affiliations:** Kasturba Medical College, Mangalore, Manipal Academy of Higher Education, Manipal, India

While the discussion is mainly on specific concepts of modality difference, nonetheless, it will be useful to understand why a larger discrepancy was noted for (gold coin, cupboard vs. traffic, key) for the group I compared to group IV (Figure 2).

- The larger discrepancy for the recall of words ‘traffic’ and ‘key across the age groups were because these two words were presented in auditory modality alone. On the contrary, the remaining two words – ‘gold coin’ and cupboard were presented in the auditory-visual modality and because of the contribution of visual modality (which is reported to have faster maturation of as young as 6 years of age) had a lesser discrepancy in recall across the age groups.
- The suggestion raised is already addressed in the last but one paragraph of the research article as a justification for the finding:
- “Lastly, Ferrara *et al.* (2017) reported that detailed visual memory capacity is present in children as young as six years of age, as a result of faster maturation of visual memory than the auditory memory. This can be considered as a contributing factor for observing no differences in auditory-visual recall performance across the age group.”

I note the following in the last para - 'These evidences supports the findings of the present study; an increase in STM skills with age,' how? The description provided in the method section does not detail about how STM was tested or tapped on.

 The quoted line has been corrected as

 “These evidences supports the findings of the present study; an increase in recall ability with age” in the third version of the article

# Peer review: Reviewer response for version 2

**DOI:** 10.5256/f1000research.29224.r71335

**Published:** 2020-10-07

**Authors:** Ramesh Kaipa

**Affiliations:** 1 Department of Communication Sciences and Disorders, Oklahoma State University, Stillwater, OK, USA

Thank you to the authors for addressing my comments. My only comment would be for the authors to address the limitations of the current study. But otherwise this papers will be a decent contribution to the existing literature.

Is the work clearly and accurately presented and does it cite the current literature?

No

If applicable, is the statistical analysis and its interpretation appropriate?

Yes

Are all the source data underlying the results available to ensure full reproducibility?

No

Is the study design appropriate and is the work technically sound?

Partly

Are the conclusions drawn adequately supported by the results?

Partly

Are sufficient details of methods and analysis provided to allow replication by others?

No

Reviewer Expertise:

Speech and Language Learning

I confirm that I have read this submission and believe that I have an appropriate level of expertise to confirm that it is of an acceptable scientific standard, however I have significant reservations, as outlined above.

# Response

**Published:** 2020-12-25

**Authors:** Malavika Anil

**Affiliations:** Kasturba Medical College, Mangalore, Manipal Academy of Higher Education, Manipal, India

The study limitations are added in the discussion section of the new version as suggested.

# Peer review: Reviewer response for version 1

**DOI:** 10.5256/f1000research.25805.r65393

**Published:** 2020-07-24

**Authors:** Raju Sapkota

**Affiliations:** 1 Vision and Eye Research Institute, School of Medicine, Anglia Ruskin University, Cambridge, UK

The major issue with this article is that the background is poorly developed. There is abundant evidence in literature on the effect of visual, auditory or cross-modal effects in STM.

 Also, while the authors associate the findings to aging effect, it could well be that the effects are merely the effect of learning, since children become more experienced learners with age.

 The findings are not discussed adequately in context of the existing literature. I suggest addressing these issues before the article is accepted for indexing.

Is the work clearly and accurately presented and does it cite the current literature?

No

If applicable, is the statistical analysis and its interpretation appropriate?

Partly

Are all the source data underlying the results available to ensure full reproducibility?

Partly

Is the study design appropriate and is the work technically sound?

Partly

Are the conclusions drawn adequately supported by the results?

Partly

Are sufficient details of methods and analysis provided to allow replication by others?

Partly

Reviewer Expertise:

Visual short-term memory, cross-modal binding, early dementia, aging effects in human visual short-term memory

I confirm that I have read this submission and believe that I have an appropriate level of expertise to confirm that it is of an acceptable scientific standard, however I have significant reservations, as outlined above.

# Response

**Published:** 2020-07-29

**Authors:** Malavika Anil

**Affiliations:** Kasturba Medical College, Mangalore, Manipal Academy of Higher Education, Manipal, India

Pictures over words: a cross-sectional study reporting short term memory abilities in children

 Rooha A, Anil MA and Bhat JS

**INTRODUCTION:**

 The major issue with this article is that the background is poorly developed. There is abundant evidence in the literature on the effect of visual, auditory or cross-modal effects in STM

**INTRODUCTION:**

- The paper was designed considering a word limit of 1000 words, hence an extensive literature review was not included in the Introduction. However, the authors have built the need by citing relevant literature to peruse the study in the introduction.

 

**DISCUSSION:**

 Also, while the authors associate the findings to the aging effect, it could well be that the effects are merely the effect of learning since children become more experienced learners with age.

**DISCUSSION:**

- While the ‘aging effect’ and ‘more learning experience with age’ can be associated with memory performance, the authors have discussed majorly on the difference in recall between the modalities. The discussion revolves around specific concepts of modality difference.

**DISCUSSION:**

 The findings are not discussed adequately in the context of the existing literature. I suggest addressing these issues before the article is accepted for indexing.

**DISCUSSION:**

- The findings are related to the concepts of existing literature. The review of the existing literature has not been added to the manuscript as the paper was designed considering a word limit of 1000 words. However, we have tried to discuss in light of major contributing studies.

# Peer review: Reviewer response for version 1

**DOI:** 10.5256/f1000research.25805.r63646

**Published:** 2020-06-18

**Authors:** Ramesh Kaipa

**Affiliations:** 1 Department of Communication Sciences and Disorders, Oklahoma State University, Stillwater, OK, USA

Introduction:

 I don’t really understand what the rationale is for the current study? The authors do not build an appropriate case for pursuing this line of study. I would like to see some extensive review of previous literature. The authors also need to mention how would the current study contribute to the empirical body of knowledge.

 Methods:

 I would like to see a detailed explanation of how the test was administered. The authors mentioned it was presented using a laptop but I would like to see specific information on how long each of the stimuli appeared on the screen. How long were the participants allowed to wait before providing a response? Was there a penalty if the participants waited for an extended period of time before they provided a response?

 Discussion

 The authors make a case that words such as “gold coins” and “cupboard” were recalled by more number of participants. I wonder is it because the participants are exposed to these stimuli items more than the other items that were presented to them? I wish the authors had performed a familiarity rating on each of the stimuli items. Currently, it is unknown what played a role in better identification of specific words over others? This is a major confound of the current study,

Is the work clearly and accurately presented and does it cite the current literature?

No

If applicable, is the statistical analysis and its interpretation appropriate?

Yes

Are all the source data underlying the results available to ensure full reproducibility?

No

Is the study design appropriate and is the work technically sound?

Partly

Are the conclusions drawn adequately supported by the results?

Partly

Are sufficient details of methods and analysis provided to allow replication by others?

No

Reviewer Expertise:

Speech and Language Learning

I confirm that I have read this submission and believe that I have an appropriate level of expertise to state that I do not consider it to be of an acceptable scientific standard, for reasons outlined above.

# Response

**Published:** 2020-07-29

**Authors:** Malavika Anil

**Affiliations:** Kasturba Medical College, Mangalore, Manipal Academy of Higher Education, Manipal, India

Pictures over words: a cross-sectional study reporting short term memory abilities in children

 Rooha A, Anil MA and Bhat JS

**INTRODUCTION:**

 I don’t really understand what the rationale is for the current study? The authors do not build an appropriate case for pursuing this line of study. I would like to see some extensive review of previous literature. The authors also need to mention how would the current study contribute to the empirical body of knowledge.

**INTRODUCTION:**

- The paper was designed considering a word limit of 1000 words, hence an extensive literature review was not included in the Introduction. However, the authors have built the need by citing relevant literature to peruse the study in the introduction.
- The contribution of the present study to the empirical body of knowledge has been discussed in the concluding paragraph of the discussion section.

 

**METHOD:**

 I would like to see a detailed explanation of how the test was administered. The authors mentioned it was presented using a laptop but I would like to see specific information on how long each of the stimuli appeared on the screen. How long were the participants allowed to wait before providing a response? Was there a penalty if the participants waited for an extended period of time before they provided a response?

**METHOD:**

- As the study focused only on the accuracy of the recall and not the reaction time measures, time-related aspects were not considered in the methodology section.

** **


**DISCUSSION:**

 The authors make a case that words such as “gold coins” and “cupboard” were recalled by more number of participants. I wonder is it because the participants are exposed to these stimuli items more than the other items that were presented to them? I wish the authors had performed a familiarity rating on each of the stimuli items. Currently, it is unknown what played a role in better identification of specific words over others? This is a major confound of the current study,

**DISCUSSION:**

- The story included words that are familiar to children, ascertained through a textbook analysis of vocabulary.
- Content validation was also done by primary school English teachers who approved the familiarity of the words considered in the story by all students.
- The words considered were all nouns, having only a single frequency of occurrence in the whole story which was also content validated by primary school English teachers and Speech-Language Pathologist.
